# Chronic Pilonidal Cyst with Malignant Transformation: A Case Report and Literature Review

**DOI:** 10.7759/cureus.23248

**Published:** 2022-03-17

**Authors:** Rachel E Pyon, Anika Mazumder, Fawwaz Almajali, Scott Wong

**Affiliations:** 1 Department of Surgery, Saint Louis University School of Medicine, St. Louis, USA

**Keywords:** chronic pilonidal sinus, pilonidal cyst surgery, pilonidal surgery, pilonidal malignancy, pilonidal, pilonidal sinus surgery

## Abstract

A 63-year-old male with a 20-year history of a chronic, recurrent sacrococcygeal pilonidal cyst was referred to our outpatient clinic. He had received multiple surgical resections in the past with benign pathology. He presented with a verrucous wart-like midline mass on the superior gluteal cleft that had grown since his last resection. The patient subsequently underwent resection of the mass with bilateral gluteal rotational flaps. Pathology showed squamous cell carcinoma with tumor-free margins, and further imaging showed no evidence of metastatic disease. It is believed chronic inflammation with subsequent genetic and impaired DNA repair mechanisms is the leading cause of malignancy. The treatment of choice for pilonidal carcinoma is surgical resection with free margins. Reconstruction methods can be utilized to repair the tissue defect. Pilonidal carcinoma has high mortality risk with surgical treatment yielding a disease-free 5-year survival rate of 55% of patients and a high recurrence rate of 50%. The role of chemoradiotherapy is currently unclear.

## Introduction

Pilonidal cysts are a common disease affecting up to five percent of the population [1 ,2]. They are usually located in the sacrococcygeal area and are often complicated by cellulitis, recurrent sinus tracts, and abscess formation [3, 4, 5]. While pilonidal cysts are generally benign, malignant transformation is rare and is thought to occur in about 0.1% of cases [1, 2, 4, 5]. There are about 100 reported cases in the literature. Malignant transformation only occurs with chronic or recurrent pilonidal cysts, with 90% of cases being squamous cell carcinoma (SCC) [1]. Basal cell carcinomas, adenocarcinomas, and verrucous carcinomas have also been reported [1, 2, 4]. Treatment involves surgical excision, with wide, tumor-free margins yielding the best outcomes [1, 4, 5]. Radiotherapy has been shown to decrease recurrence rates, but the role of adjuvant chemotherapy is unclear [4]. We report an extremely rare case of SCC developing in a 63-year-old male with a chronic recurrent sacrococcygeal pilonidal cyst.

## Case presentation

A 63-year-old male with chronic, recurrent sacrococcygeal pilonidal cyst was referred to our outpatient clinic for evaluation and treatment. He had a 20+ year history of recurrent pilonidal disease requiring multiple prior excisions, with the most recent resection with local flap closure done five years ago. Pathology at that time was benign. Since then, his pilonidal cyst has recurred, grown, and is characterized by pain and purulent and sanguineous drainage. He has required multiple debridements of the pilonidal cysts with ACell MatriStem (ACell, Columbia, Maryland) matrix placement. His other past medical history is significant for hypertension, atrial fibrillation, and lymphedema requiring vascularized lymph node transfer. 

**Figure 1:**
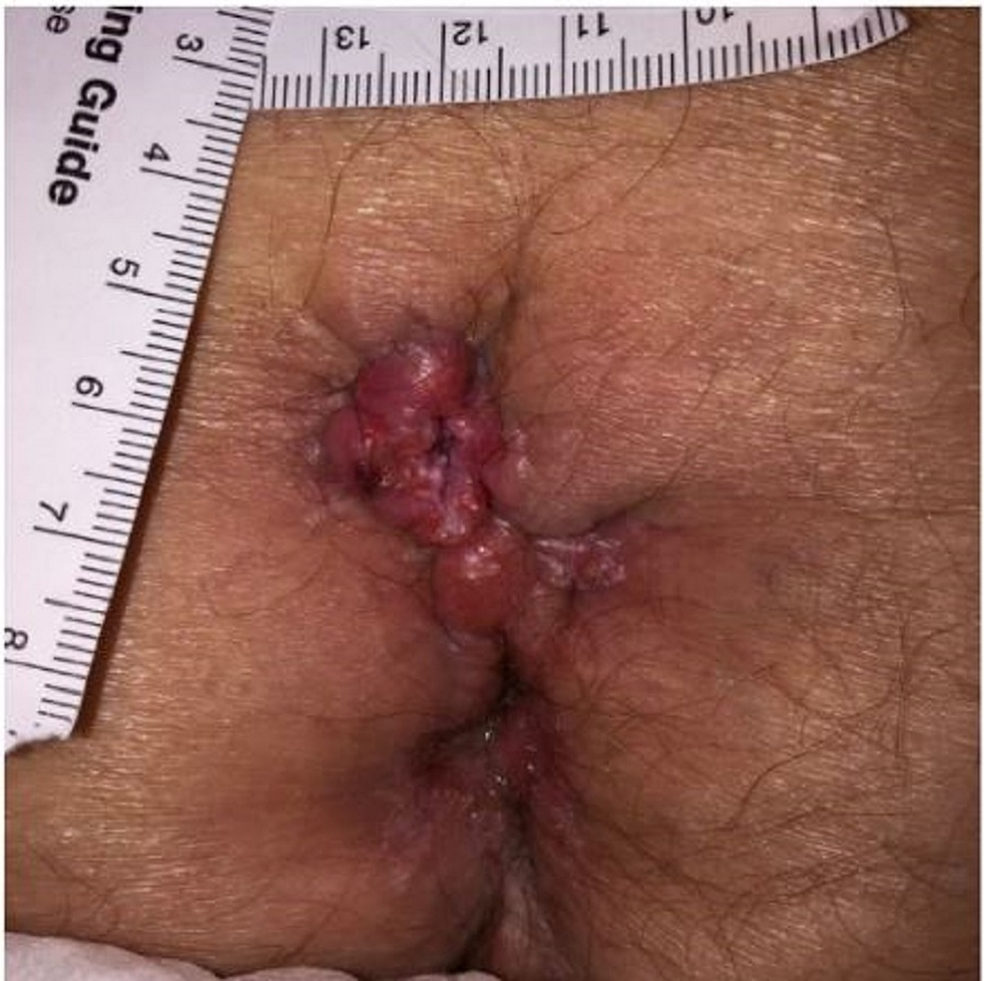
Initial presentation of chronic, recurrent pilonidal cyst

Clinical examination revealed a 1.7x1.2 cm pilonidal cyst with two open sinus tracts near the superior gluteal cleft with purulent discharge and a pilonidal pit with drainage at the midline of the intergluteal cleft (Figure 1). 

**Figure 2:**
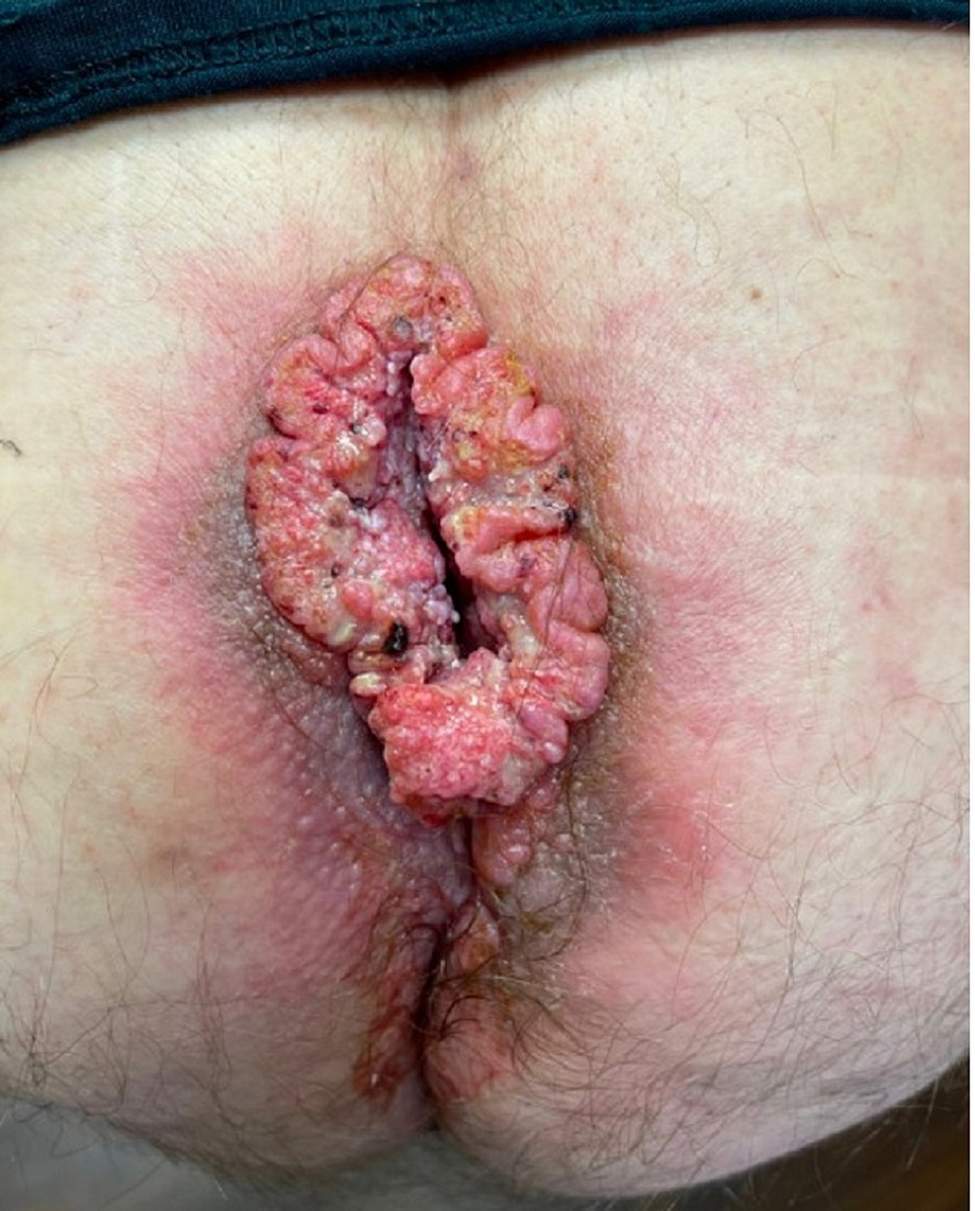
Growing verrucous anal squamous cell carcinoma

In preparation for a repeat pilonidal cyst excision and flap closure, a temporary diverting loop colostomy was recommended due to the proximity of the expected incision site to the anal verge. However, he failed to follow up and presented 10 months later and underwent a laparoscopic diverting loop colostomy with no complications. Prior to the cyst excision and flap closure, the patient reported that the pilonidal cyst has continued to grow and drain more frequently. At that time, clinical examination revealed 8x4x4cm verrucous wart-like midline mass on the superior aspect of the rectum that tracks deeply (Figure 2). The patient underwent an excision skin biopsy of the mass, and histopathological examination revealed squamous cell carcinoma. CT of the chest, abdomen, and pelvis with contrast was negative for metastatic disease.

**Figure 3:**
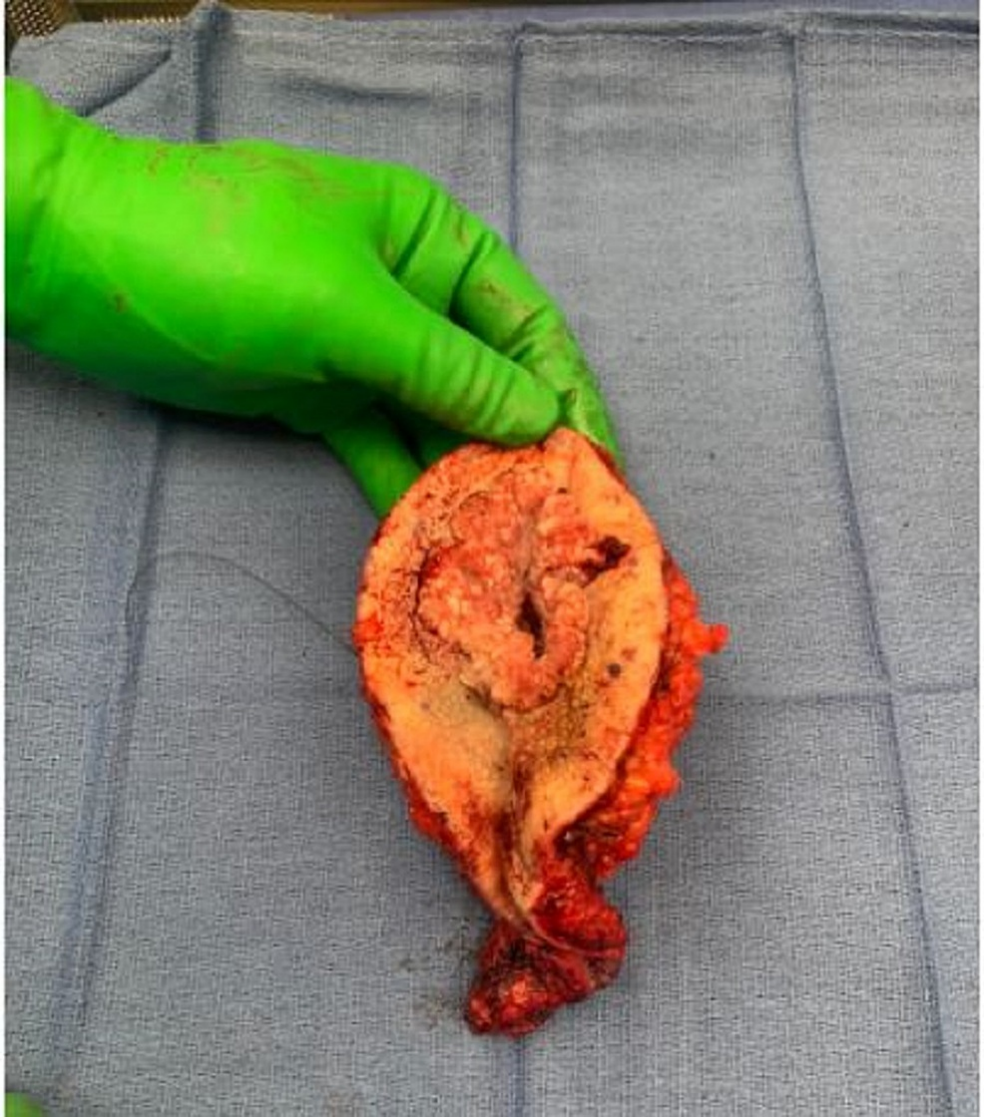
Surgical excision of the pilonidal cyst with tumor-free margins

**Figure 4:**
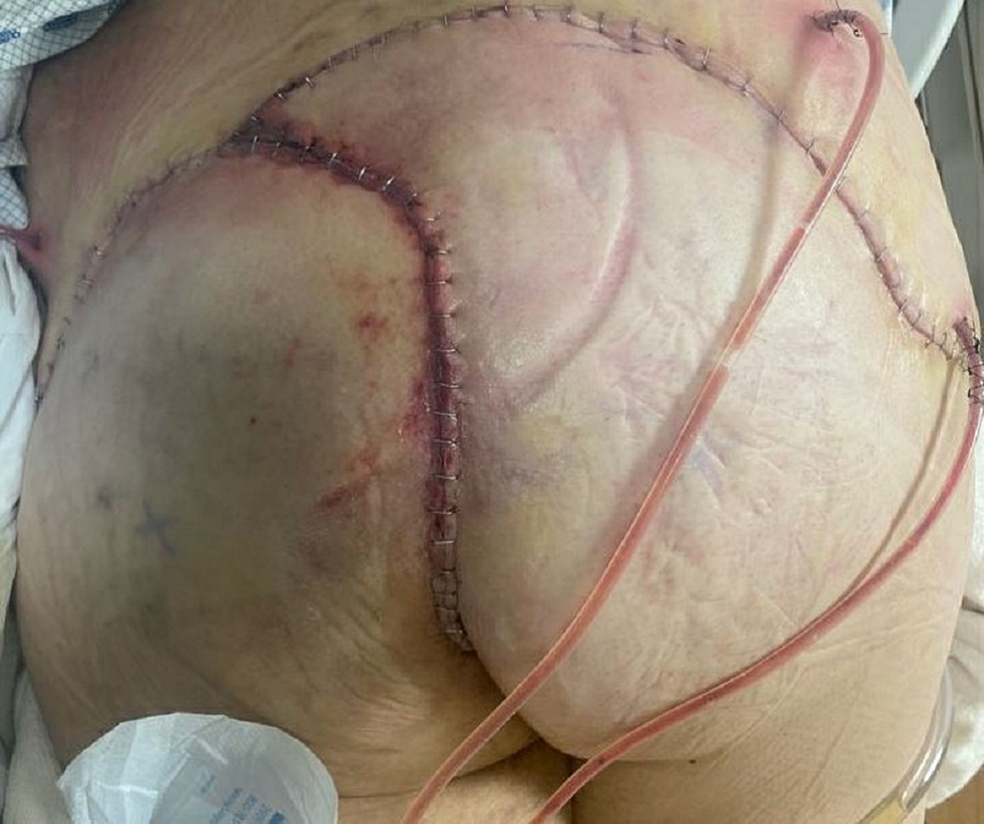
Complex pilonidal cystectomy with bilateral gluteal rotation flap reconstruction, postoperative day one

The patient underwent complex pilonidal cystectomy with bilateral gluteal rotation flap reconstruction (Figure 3, 4). Histopathological examination was significant for well-differentiated invasive squamous cell carcinoma, with the tumor-free margins of the resected mass. 

**Figure 5:**
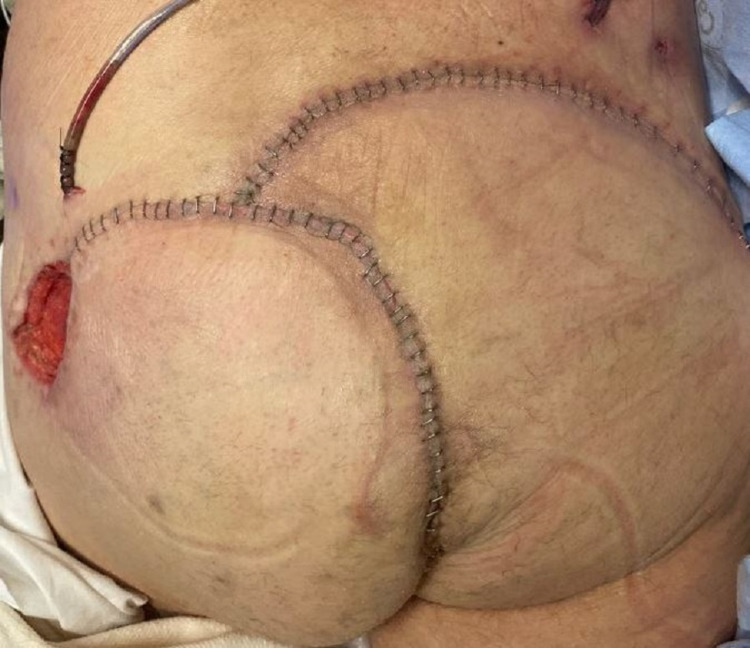
Postoperative day seven from repeat I&D I&D - irrigation and debridement

On postoperative day seven, the patient became febrile and had frank purulence at the perianal incision and drain sites. He was started on broad-spectrum antibiotics. Drain cultures grew heavy *Morganella morganii*, *Streptococcus anginosus*, *Helcococcus kunzii*, and *Proteus mirabilis*. On postoperative day 10, a CT scan of the abdomen and pelvis with contrast showed a fluid collection in the left gluteal subcutaneous tissue, and the patient underwent irrigation and debridement (I&D) of the gluteal flaps with wound vacuum placement and replacement of the drains. Four days later, the patient underwent a repeat I&D of the gluteal flaps with a delayed partial closure of the bilateral gluteal fasciocutaneous flaps, left gluteal, and incisional wound vacuum placement, and sacral bone biopsy and culture. Bone cultures grew *Morganella morganii *with pathology consistent with acute osteomyelitis. The patient was continued on IV cefepime and metronidazole. On postoperative day seven from repeat I&D, the wound continued to improve with healthy granulation tissue (Figure 5). The patient was discharged with IV antibiotics and a wound vacuum.

## Discussion

Pilonidal disease is a fairly common condition diagnosed in around 70,000 patients in the United States each year [6]. Initial presentation of pilonidal disease can appear as a simple cyst, an acute abscess with or without cellulitis, or a chronically draining sinus [6, 7]. Pilonidal cysts are most commonly found in the sacrococcygeal region about 3 to 5 cm superior to the anus and are believed to arise due to loose hair in the natal cleft that causes a foreign body reaction with subsequent chronic inflammation [8]. Risk factors for the development of pilonidal disease include obesity, sedentary lifestyle, family history, hirsute body habitus, and trauma or irritation to the gluteal cleft skin [6]. Untreated pilonidal cysts can further lead to complications such as cellulitis, chronic infection, and abscess formation. 

Although uncommon, pilonidal disease can transform into carcinoma similarly to that of other chronically inflamed wounds such as osteomyelitis, scars, skin ulcers, and fistulas [3]. It is believed that this process is a culmination of free oxygen radical release from activated inflammatory cells that cause genetic damage, neoplastic inflammation, and impaired DNA repair mechanisms leading to malignancy [8]. Although the literature often quotes the occurrence rate of malignant degeneration is about 0.1% of pilonidal cyst cases, the actual incidence is not known, with the most common histologic type being squamous cell carcinoma in 88% of cases. Gross clinical signs of this transformation can include a growing, ulcerated mass usually >5 cm in diameter with indurated edges [9]. In addition, inguinal adenopathy is an uncommon but poor prognostic sign in pilonidal carcinoma associated with a survival time of only seven months [10].

Diagnosis of malignant transformation of pilonidal cysts is confirmed by biopsy. The most crucial consideration in the histological differential diagnosis of pilonidal carcinoma is pseudocarcinomatous hyperplasia of squamous epithelium associated with a severe inflammatory process [9, 10]. Furthermore, metastasis to the intra-abdominal region such as the iliac and para-aortic nodes can be assessed by physical exam for lymphadenopathy and computerized tomography or MRI. The presence and extent of distant metastasis should be assessed utilizing positron emission tomography [1, 2].

The treatment of choice in pilonidal carcinoma is surgical resection with tumor-free margins. As the tumor may extend along fistula tracts in a wide area of the sacrococcygeal and perineal regions, resection must include at least the presacral fascia, but often also portions of the sacrum, coccyx, and rectum [8]. Following surgical excision, various flap-based reconstruction methods can be utilized to repair the resultant tissue defect depending on the size and depth of the defect and includes the Bascom Cleft Lift, Z-plasty, W-plasty, V-Y fasciocutaneous flap, rhomboid, gluteus maximus rotational flap, and Dufourmentel flap [6]. There is one reported case of closure by secondary intention, which was chosen due to an extensive defect, onset of the infectious process, and hemodynamic instability [5]. However, closure by primary intention has been reported to provide earlier wound healing, shorter hospital stay, and higher quality of life after surgery [11].

Unfortunately, pilonidal carcinoma has a poor prognosis with surgical treatment yielding a disease-free five-year survival rate of 55% of patients and a high recurrence rate in more than half of patients [12]. Therefore, clinical suspicion warrants early investigation, and histologic suspicion should prompt repeat wide local excision with detailed pathologic exam. Notably, combining radical surgery with radiotherapy appears to reduce local recurrence rates to 30% compared to 50% with only surgical resection [2, 12]. In comparison, the role of chemotherapy is currently unclear but may be considered in high-risk lesions (lymphovascular/perineural invasion, positive margins) in combination with surgery and radiotherapy [2].

## Conclusions

Pilonidal cysts are a benign condition that has the potential for malignant transformation through a chronic inflammatory process. When patients with chronic pilonidal disease present with poor wound healing, chronic fistulas, or growing ulcerated masses, malignant transformation should be suspected. We recommend that all specimens of pilonidal disease be sent for histopathological investigation, especially given the high mortality rate of SCC in pilonidal disease cases. Following the diagnosis of SCC, treatment should consist of surgical excision with free margins, flap closure based on the size of the lesion, and radiotherapy to reduce recurrence rates. 
